# Prospective cohort study on the predictors of fall risk in 119 patients with bilateral vestibulopathy

**DOI:** 10.1371/journal.pone.0228768

**Published:** 2020-03-09

**Authors:** Bieke Dobbels, Florence Lucieer, Griet Mertens, Annick Gilles, Julie Moyaert, Paul van de Heyning, Nils Guinand, Angelica Pérez Fornos, Nolan Herssens, Ann Hallemans, Luc Vereeck, Olivier Vanderveken, Vincent Van Rompaey, Raymond van de Berg

**Affiliations:** 1 Faculty of Medicine and Health Sciences, University of Antwerp, Antwerp, Belgium; 2 Department of Otorhinolaryngology & Head and Neck Surgery, Antwerp University Hospital, Edegem, Belgium; 3 Department of Otorhinolaryngology and Head and Neck Surgery, Division of Balance Disorders, Faculty of Health Medicine and Life Sciences, Maastricht University Medical Center, School for Mental Health and Neuroscience, Maastricht, Netherlands; 4 Service of Otorhinolaryngology Head and Neck Surgery, Department of Clinical Neurosciences, Geneva University Hospitals, Geneva, Switzerland; 5 Department of Rehabilitation Sciences and Physiotherapy/Movant, Faculty of Medicine and Health Science, University of Antwerp, Belgium; Multidisciplinary Motor Centre Antwerp (M^2^OCEAN), University of Antwerp, Antwerp, Belgium; 6 Faculty of Physics, Tomsk State Research University, Tomsk, Russia; University of Rochester, UNITED STATES

## Abstract

**Objectives:**

To identify predictive factors for falls in patients with bilateral vestibulopathy (BV). Specific variables contributing to the general work-up of a vestibular patient were compared between BV patients experiencing falls and those who did not.

**Design:**

Prospective multi-centric cohort study.

**Setting:**

Department of Otorhinolaryngology & Head and Neck Surgery at two tertiary referral centers: Antwerp University Hospital and Maastricht University Medical Center.

**Participants:**

In total, 119 BV patients were included. BV diagnosis was defined in accordance with the diagnostic BV criteria, established by the Bárány Society in 2017.

**Main outcome measures:**

Patients were divided into fallers and non-fallers, depending on the experience of one or more falls in the preceding 12 months. Residual vestibular function on caloric testing, rotatory chair testing, video head impulse test (vHIT) and cervical vestibular evoked myogenic potentials (cVEMP) was evaluated as a predictive factor for falls. Furthermore, hearing function (speech perception in noise (SPIN)), sound localization performance, etiology, disease duration, sport practice, scores on the Dizziness Handicap Inventory (DHI) and the Oscillopsia Severity Questionnaire (OSQ) were compared between fallers and non-fallers.

**Results:**

Forty-five (39%) patients reported falls. In a sub-analysis in the patients recruited at UZA (n = 69), 20% experienced three or more falls and three patients (4%) suffered from severe fall-related injuries. The DHI score and the OSQ score were significantly higher in fallers. Residual vestibular function, SPIN, sound localization performance, etiology, disease duration, age and sport practice did not differ between fallers and non-fallers.

**Conclusions:**

Falls and (severe) fall-related injuries are frequent among BV patients. A DHI score > 47 and an OSQ score > 27.5 might be indicative for BV patients at risk for falls, with a sensitivity of 70% and specificity of 60%. Residual vestibular function captured by single vestibular tests (vHIT, calorics, rotatory chair, cVEMP) or by overall vestibular function defined as the number of impaired vestibular sensors are not suitable to distinguish fallers and non-fallers in a BV population.

## Introduction

Bilateral vestibulopathy (BV) is characterized by a bilateral loss of function of the peripheral vestibular organs, the vestibular nerves or both [1]. The most frequent reported etiologies of BV are ototoxic (aminoglycosides antibiotics), Menière’s disease and infections [2–4]. In Belgium and the Netherlands a relative higher proportion of genetic etiology is found due to a COCH mutation causing DFNA9 disease, which is the most common type of autosomal dominant non-syndromic deafness [5]. However, in about half of all BV patients no underlying etiology can be identified that caused their vestibular loss [2, 6]. In these patients, the disease is called ‘idiopathic BV’. BV patients typically suffer from imbalance worsening in the dark or on uneven ground, reflecting the need of other sensory inputs to maintain postural control. Due to failure of the vestibulo-ocular reflex, BV patients might also complain of instable gaze during head movements, called oscillopsia [1].

Postural control is guaranteed by appropriate motor responses based on the multisensory integration of vestibular, visual, auditive and proprioceptive information. Due to the reduced vestibular input, this complex process is hindered in BV patients [6, 7]. The ability to detect gravity decreases and fast postural corrections become difficult. Together with oscillopsia, this exposes BV patients to an increased risk of falling. Compared to healthy peers, BV patients are reported to have a 31-fold increased risk of falling [8]. In a study assessing fall risk in patients with vertigo, BV patients were found to have the highest risk of falling amongst patients with peripheral vestibular syndromes [9].

Falling is considered one of the most important complications of BV as it results in fall-related injuries and loss of independence [6]. Moreover, the secondary induced fear of future falls can result in further social isolation and a substantial decline in quality of life [10].

As symptoms and complains vary widely in a BV population [7], this study aims to identify clinical risk factors to predict the risk of falling in BV patients, enabling clinicians to provide better and more individualized counseling of BV patients. Moreover, specialized physiotherapeutic regimens and fall prevention might reduce the incidence of falls and its burden in the BV population. Finally, given the prospect of a vestibular implant [11], this study might be helpful to highlight objective measures that could facilitate patient selection for a vestibular implant (VI).

This study differs from previous studies because it evaluates specifically those variables that are generally included in the work-up of a vestibular patient, and because of its unprecedented large sample size of BV patients (n = 119).

As different variables will be examined, the results and discussion sections have been divided into the following parts:

1. *Vestibular tests and fall risk*. Progress in vestibular testing has allowed evaluation of different frequency ranges of the lateral canal function (from low to high frequency: calorics, rotatory chair testing, video head impulse test (vHIT)). According to the diagnostic criteria of the Bárány Society [1], only one of these tests must be impaired in order to diagnose BV. Therefore, BV patients express a wide variability in their pattern of vestibular impairment. Whereas some BV patients suffer from a severely reduced lateral canal function in all tested frequencies, others only show a reduced function in, for example, the low frequencies, measured by calorics. Moreover, the function of the other semicircular canals (i.e. anterior and posterior) and the otolith organs may vary between BV patients. It remains unclear whether the degree of vestibular impairment correlates with the incidence of falls.
2. *Hearing performance and fall risk*. BV patients frequently suffer from an associated hearing loss. Keeping balance is a complex process relying on not only vestibular input, but on other sensory information as well (proprioception, vision, hearing, etc…) [12]. Patients suffering from hearing loss are at a disadvantage to use external sounds as spatial landmarks [12]. We therefore hypothesized that BV patients with worse hearing performance and worse sound localization performance are at a higher risk of falling.
3. *Disease/patient’s characteristics and fall risk*. Animal studies have demonstrated that within 10 days after the onset of BV, the non-labyrinthine inputs to the vestibular nuclei are rapidly amplified [13]. The process of reweighing other sensory input systems to maintain balance, is called central compensation [14]. We hypothesized that the more a patient has compensated, the less likely it is that falls will occur. Therefore, a shorter duration of BV disease could be a risk factor for falling. Furthermore, we aimed to evaluate whether sport practice, which is believed to facilitate the central compensation [15], is a preventive factor for falls.
4. *Symptom questionnaires and fall risk*. The Dizziness Handicap Inventory (DHI) assesses the subjective experience of dizziness and its impact on quality of life, whereas the Oscillopsia Severity Questionnaires (OSQ) specifically addresses the symptoms of oscillopsia. Both questionnaires are easy to administer prior to the outpatient’s visit. When correlating with the risk of falling of a BV patient, the scores on these questionnaires might be useful to help the clinician identifying those patients at a high risk of falling.

## Methods

### Study design

The current study was a two-center, prospective, cohort study, recruiting from October 2017 until February 2019 at the Antwerp University Hospital (UZA) and from February 2016 until July 2018 at the Maastricht University Medical Center (MUMC+). The study was approved by the local ethics committees of the UZA (protocol number 16/42/426) and of the MUMC+ (protocol number NL52768.068.15 / METC 151027). The study was registered on ClinicalTrials.gov (UZA, (NCT03690817)) and trialregister.nl (MUMC+, Trial NL5446 (NTR5573)). All patients provided a written informed consent before enrollment in the study.

### Study participants

A total of 119 BV patients were included in this study, of which 69 were recruited from the Otorhinolaryngology, Head and Neck surgery department at UZA and 50 patients at MUMC+. Only patients who met the BV diagnostic criteria, recently established by the Bárány Society, could participate in this study [1]:

1. horizontal angular vestibulo-ocular-reflex (VOR) gain < 0.6 measured by the video head impulse test (vHIT), and/or
2. reduced caloric response (sum of bithermal, 30° and 44°, max. peak slow phase velocity (SPV) on each side < 6°/sec), and/or
3. reduced horizontal angular VOR gain < 0.1 upon sinusoidal stimulation on a rotatory chair.

### Risk of falling

Patients received a questionnaire in which they were asked about falls in the preceding 12 months. This questionnaire was orally discussed with the patient in an interview until all ambiguities were clarified. Based on the following question, patients were divided into two groups, fallers and non-fallers: ‘Have you fallen in the past year due to slipping or tripping, losing balance thereby ending on the floor or another lower level?’. Furthermore, patients recruited at UZA were asked about (1) how many times they had fallen, (2) where they had fallen, (3) what caused them to fall and (4) fall-related injuries.

### Vestibular testing

By enrollment in the study, all BV patients received neuro-otological testing on site. A multifrequential evaluation of the function of the lateral semicircular canal was performed:

- Caloric testing (low-frequency function):
  Bithermal caloric irrigation (30°/44°) was performed with the patient in supine position with a head-incline of 30°. Nystagmus was recorded using electronystagmography (ENG).
- Rotatory chair testing (low- to mid-frequency function):
  Patients were seated in a servo-controlled rotatory chair in the dark. Nystagmus was recorded using electronystagmography (ENG). Rotatory chair tests were performed using sinusoidal rotation (0.05 Hertz in UZA and 0.1 Hertz in MUMC+) with a peak velocity of 60°/sec [16]. More detailed methodology and normative data have previously been described [16].
- Video head impulse testing (vHIT) (high-frequency function):
  All impulses were performed by experienced examiners. Angular head velocity was determined by three mini-gyroscopes, eye velocity by means of an infrared camera recording the right eye, all incorporated in commercially available vHIT goggles (Otometrics, Taastrup, Denmark). Vestibulo-ocular reflex (VOR) gain was defined as the ratio of the area under the eye velocity curve to the head velocity curve from the impulse onset until the head velocity dropped to zero again [17]. A minimum of ten valid impulses was required in each canal direction. Lateral impulses were realized with a target speed >200°/s and vertical impulse with a target speed >150°/s.

In addition, the left anterior and right posterior canal were assessed during vHIT. First, the head was turned 40° to the right and then impulses were given with a pitch rotation in the plane of the canals. Likewise, the head was rotated 40° to the left to assess the function of the right anterior and left posterior canal with similar impulses.

Additionally, saccular function was evaluated by performing c-VEMP testing. Details on the procedure have been published previously [18, 19]. In brief, a patient’s saccular function was quantified by the response of the ipsilateral sternocleidomastoid muscle to air-conducted 500 Hz tone bursts delivered monoaurally via insert phones. Recordings were made with an auditory evoked potential system equipped with electromyographic software (Neuro-Audio, Difra, Belgium), with self-adhesives electrodes (Blue sensor, Ambu, Denmark). The presence of a typically biphasic shape, with a positive peak after 13 ms (p13) and a negative peak after 23 ms (n23), was evaluated. In a stepwise procedure, the lowest threshold with a still present p13n13 wave response was searched. When no p13n23 wave was seen above 95 dB HL acoustic clicks, a patient was considered to have an absent cVEMP response.

### Hearing assessment

#### Speech audiometry in noise (SPIN), best-aided conditions

Given the frequently associated hearing loss in BV patients, hearing performance was evaluated in the best-aided situation in which patients could wear their normal hearing aids. Assessment was conducted in free field in an audiometric sound isolated booth using the Leuven Intelligibility Sentences Test (LIST) (van Wieringen and Wouters 2008) [20]. This speech material consisted of several lists of ten sentences. An adaptive procedure was used to determine the speech reception threshold (SRT). The level of the speech-weighted noise was fixed at 65 dB SPL and the intensity level of the sentences varied in steps of 2 dB adaptively in a one-down, one-up procedure according to participant’s response. The SRT was ascertained based on the level of the last 6 sentences of 2 lists, including an imaginary 11th sentence. A lower SRT indicates better speech in noise perception and thus better hearing function. The average SRT in anormal hearing population is -7.8 ± 1.17 dB speech-to-noise ratio [20].

#### Sound localization test (best-aided)

**Fig 1:**
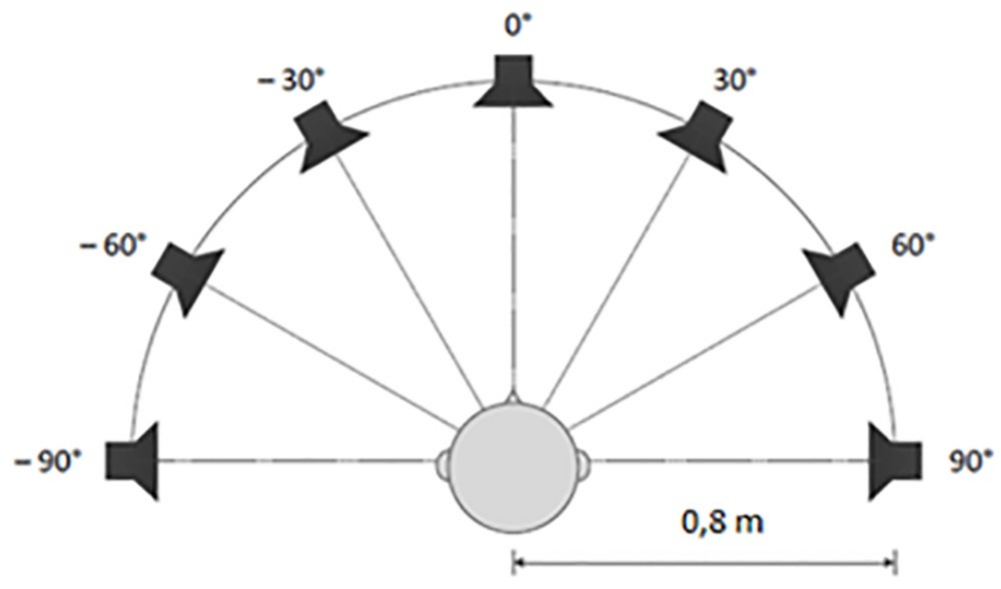
Sound localization test set-up. Seven Broadband Fostex 6301 loudspeakers at intervals of 30°, located in a frontal horizontal semicircle at the subject’s head level.

Sound source identification was evaluated in a sound-isolated booth. Again, to mimic daily life situations patients could wear their normal hearing aids. Seven loudspeakers were located in a frontal semicircle in a horizontal plane at the level of the patient’s head (Fig 1). Broadband noise stimuli were roved by +/- 5dB (sound levels between 65–75 dB SPL). All stimuli had a 200-ms duration and were delivered via one of the seven speakers in a random sequence. In total, 21 stimuli were delivered, three by every speaker. During testing, the head of the patient was directed forward in the direction of speaker at 0°. Head movement was prohibited during stimulus presentation, which was controlled by the examiner. After each stimulus, the participant had to answer from which speaker the sound was presented. No feedback about the performance was given. For each of the 42 stimuli, the angular error, i.e. the angle difference between the active speaker and the recognized speaker, was recorded. Patient’s accuracy of sound localization was evaluated via the root mean square localization error (RMSE). The higher the RMSE, the more incorrect judged azimuth’s and thus the worse the accuracy of sound localization. More detailed information on the test settings and calculation of the RMSE have previously been described [21].

### Symptom questionnaires

Questionnaires were sent to all patients to be filled out prior to the study visits. At the start of the test visit, the examiner went through all questionnaires with the patient and possible ambiguities were clarified.

#### Dizziness Handicap Inventory (DHI)

The DHI is a well-known validated questionnaire, designed to assess the self-perceived effect of dizziness on quality of life. It consists of 25 items, which are grouped into 3 subscales evaluating the respondent’s performance along emotional, functional and physical aspects of daily life. The higher the score, the more symptoms a patient experiences (range 0 to 100) and the lower the quality of life [22]. Within a range from 30 to 60 DHI scores, a moderate self-perceived handicap is present. DHI scores above 60 point at a severe handicap [23].

#### Oscillopsia severity questionnaire (OSQ)

This questionnaire was developed for assessing the severity of oscillopsia in a BV population by Guinand and colleagues [4]. Nine different daily life situations are described and the experience of oscillopsia is formulated as a ‘sensation that the visual environment is moving when it’s not’. The response on each item was scored as follows: 1 (never), 2 (seldom), 3 (sometimes), 4 (often), or 5 (always). Hence, the higher the score, the higher the self-perceived frequency of oscillopsia (range 9 to 45).

#### Hospital Anxiety and Depression Scale (HADS)

In order to quantify depression and anxiety, the HADS was administered. The HADS is a widely used instrument for detecting states of depression and anxiety [24]. Both subscales, Depression and Anxiety, consist of seven items which are scored from 0 to 3. E.g. ‘I feel restless and have to be on the move’ (item relating to Anxiety subscale), ‘I can laugh and see the funny side of things’ (item relating to Depression subscale).

#### General questionnaire

Additionally, in a semi-structured interview, the following topics were covered: hearing aids, weight, length, sport practice and fall incidence during the preceding year (S3 and S4).

### Data collection and statistics

Data were stored in OpenClinica LLC (Waltham, MA, USA), an online database for electronic data registration and data management developed for clinical research. IBMS SPSS Statistics 24 (IBM; Armonk, NY) was used for the statistical analyses. Patients were divided in two groups: fallers and non-fallers. Subsequently, differences in the described variables were analyzed using independent t-test (continuous data), chi-squared test (categorical data), or the appropriate non-parametric tests. The following data were continuous date: caloric response (sum SPV), gain rotatory chair testing, vHIT gains, age, disease duration, hours of sport practice, BMI, RMSE, SRT, DHI scores, HADS scores and OSQ scores. On the other hand, fall group (fallers vs non-fallers), etiology and gender were classified as nominal categorical data. Overall vestibular function, defined as number of impaired vestibular tests and cVEMP response (bilateral absent, unilateral absent, bilateral present) were categorized as ordinal categorical data. When appropriate, Bonferroni Holm corrections were made.

## Results

### Study population

In total, 119 BV patients were included (69 patients at UZA and 50 patients at MUMC). The mean age was 59 ± 12 years (range: 21–83 years), 64 patients (54%) were male. The most frequent underlying causes were DFNA9 disease (n = 20, 17%), infectious etiology (n = 13, 11%), ototoxicity (n = 13, 11%) and Menière’s disease (n = 6, 5%). In half of the patients (n = 59, 50%) no underlying etiology could be identified. Thirty-nine percent of all patients reported a fall event in the last year. For further analyses, the patients were divided into fallers (n = 45) and non-fallers (n = 71). Three patients could not reliably remember fall events, hence they were excluded for the analyses.

### Fall details

In the patients recruited at the UZA (n = 69), more detailed information was asked about the falls, see Table 1. From this subset of 69 BV patients, 60% (n = 39) reported no falls, 12% (n = 8) one fall, 12% (n = 8) two falls and 20% (n = 14) three or more falls in the preceding year. Patients most frequently reported falls related to taking the stairs, getting on/of a chair, getting in/out of the shower or bath and ambulation on even ground in their own house. Most of the time falls were caused by losing balance (n = 23). In the majority of BV patients, no severe fall-injuries were reported. However, in one patient a fall was complicated by a hip fracture and in two patients by rib fractures.

**Table 1:** Detailed information on falls in the 69 patients at the Antwerp University Hospital (UZA). Thirty of these BV patients had experienced falls in the preceding year.

**Frequency of falls in the past year**		**n**	**Cause of fall**	**n**
None		39	Tripping	8
One		8	Slipping	7
Two		8	Loss of balance	23
Three or more		14	Dizzy	7
**Place of falls**		**n**	**Fall related injuries**	**n**
In patient’s house, related to:		Total = 46	Total	33
	*Toilet*	3	*Bruises*	*17*
	*Bath/douche*	8	*Scrapes*	*8*
	*Stairs*	11	*Hip fracture*	*1*
	*Bed*	5	*Rib fracture*	*2*
	*Flat surface*	11	*Backache*	*5*
	*Chair*	8		
In patient’s garden		Total = 40		
Outdoor, related to:		Total = 18		
	*Someone else’s house*	5		
	*Public building*	6		
	*Car*	2		
	*Street gutter*	5		

### Vestibular tests and fall risk

#### Semicircular canal function

Results of calorics, rotatory chair testing and vHIT are summarized in Table 2. Lateral canal function did not seem to differ between fallers and non-fallers, neither in its low to mid frequencies (calorics, rotatory chair), nor in its high frequencies (vHIT). There was no statistically significant difference in the function of the right versus left lateral canal at any of the tested frequencies (calorics, vHIT). Therefore, we additionally evaluated whether the bilateral sum of the lateral canal function was different between the fallers and non-fallers. Lower vHIT gains of the lateral canal were found in the non-faller group (0.76 ± 0.5 versus 0.85 ± 0.5 in the faller group). Likewise, the bilateral caloric response was more reduced in the non-faller group (2.46 ± 4.2 °/s versus 2.88 ± 1.1 °/s in the faller-group). Yet, none of these differences were statistically significant. Similarly, there was no evidence that anterior or posterior canal function had an influence on fall risk. Also, the total semicircular canal function assessed by the vHIT (sum of all gains) did not differ significantly between the fallers and non-fallers.

**Table 2:** Results of caloric, rotatory chair and vHIT testing.

	Fallers (n = 45)	Non-fallers (n = 71)	Statistics	
			Without correction multiple testing	Bonferroni Holm correction
**Video HIT**				
gain right lateral	0.43 ± 0.3	0.39 ± 0.3	p > 0.05	
gain left lateral	0.43 ± 0.3	0.37 ± 0.2	p > 0.05	
gain right posterior	0.44 ± 0.2	0.34 ± 0.2	p > 0.05	
gain left posterior	0.48 ± 0.2	0.39 ± 0.2	p > 0.05	
gain right anterior	0.53 ± 0.2	0.51 ± 0.3	p > 0.05	
gain left anterior	0.53 ± 0.2	0.44 ± 0.2	p > 0.05	
sum gain lateral canals	0.85 ± 0.5	0.76 ± 0.5	p > 0.05	
sum gain anterior canals	1.07 ± 0.4	0.95 ± 0.4	p > 0.05	
sum gain posterior canals	0.92 ± 0.4	0.74 ± 0.4	p = 0.04	p = 0.1
sum gain all canals	2.88 ± 1.1	2.46 ± 1.2	p > 0.05	
**Calorics**				
sum SPV bilateral bithermal	3.2 ± 6.8	2.4 ± 4.2	p = 0.9	
**Rotatory chair test**				
sum SPV bilateral bithermal	0.09 ± 0.1	0.09 ± 0.1	p = 0.5	

#### Saccular function

**Fig 2:**
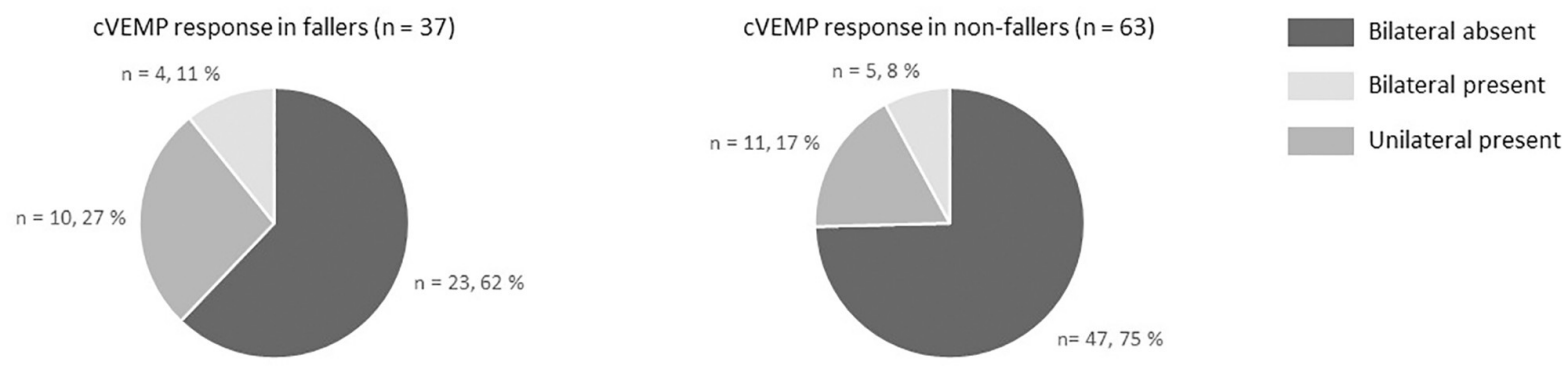
Saccular function assessed by the cervical VEMP.

In 71 patients (70%) no cVEMP response could be evoked bilaterally. In 21 patients (21%) a unilateral cVEMP response was present, and in 10 patients (10%) bilateral cVEMP responses were found. In 17 patients, cVEMP testing was not reliable due to fatigue of the sternocleidomastoid muscle. Chi-squared test did not show any significant differences between saccular function and fall incidence (p = 0.5). As can be seen from Fig 2, the proportion of patients with a bilateral absent cVEMP response was higher in the non-fallers.

#### Overall vestibular function (impaired vestibular tests)

**Fig 3:**
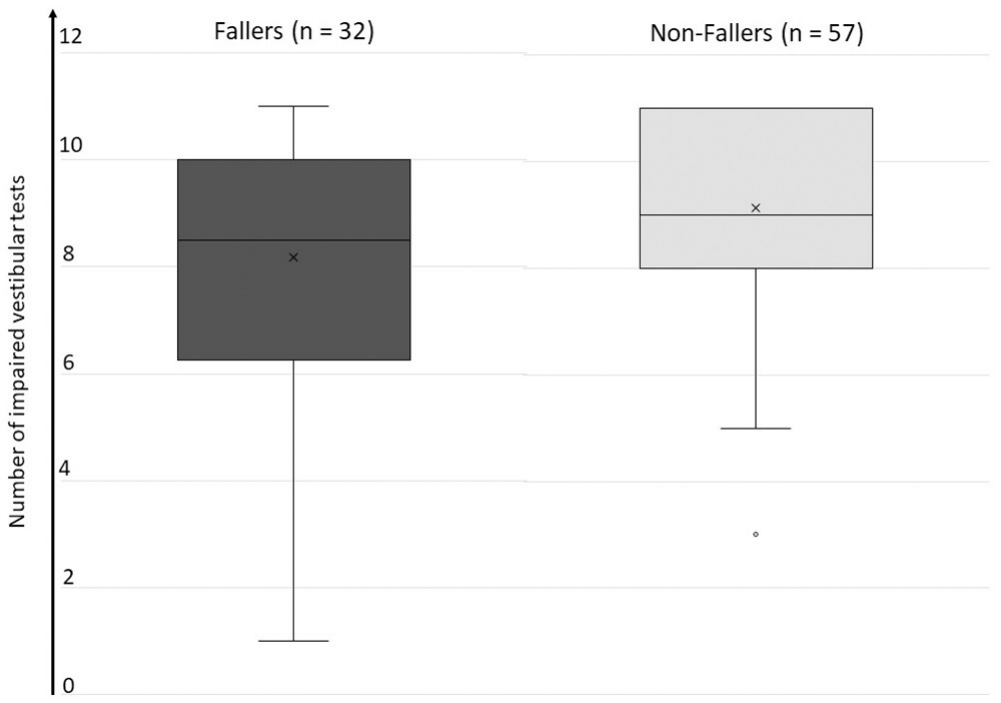
Overall vestibular function, defined as the number of impaired vestibular tests. There was not a statistically significant (p = 0.054) lower vestibular function in the non-faller group. Included vestibular tests were video head impulse test of all canals (impaired if gain ≤ 0.6), rotatory chair test (impaired if gain ≤ 0.1), calorics in each ear (impaired if sum max. SPV ≤ 6°/s) and cVEMP (impaired if absent response). A number of 12 impaired vestibular tests thus represents the least residual vestibular function.

The overall vestibular function was categorized by the number of impaired vestibular tests, thus the higher, the less residual vestibular function (range 1 tot 11). Surprisingly, the group of the non-fallers tended to have less residual vestibular function than the group of the fallers (number of impaired sensors: 9.1 ± 2 versus 8.2 ± 2.4 respectively), see Fig 3. This difference was nearly significant (p = 0.054, Mann-Whitney U test).

### Hearing performance and fall risk

SPIN and sound localization testing were performed in patients included at UZA (n = 69). In a normal hearing population the range of SRT is between -10.14 and -5.46 dB speech-to-noise ratio (SNR) [20]. Sixty patients (90%) had an SRT higher than -5.46 dB SNR, indicating impaired hearing performance. Mean SRT was 1.44 ± 6.9 dB SNR (range -7 to 20). No significant difference was found between the SRT of the fallers and non-fallers (1.99 ± 7.7 dB SNR versus 1.09 ± 6.4 dB SNR respectively, p = 0.8 in Mann-Whitney U test).

**Fig 4:**
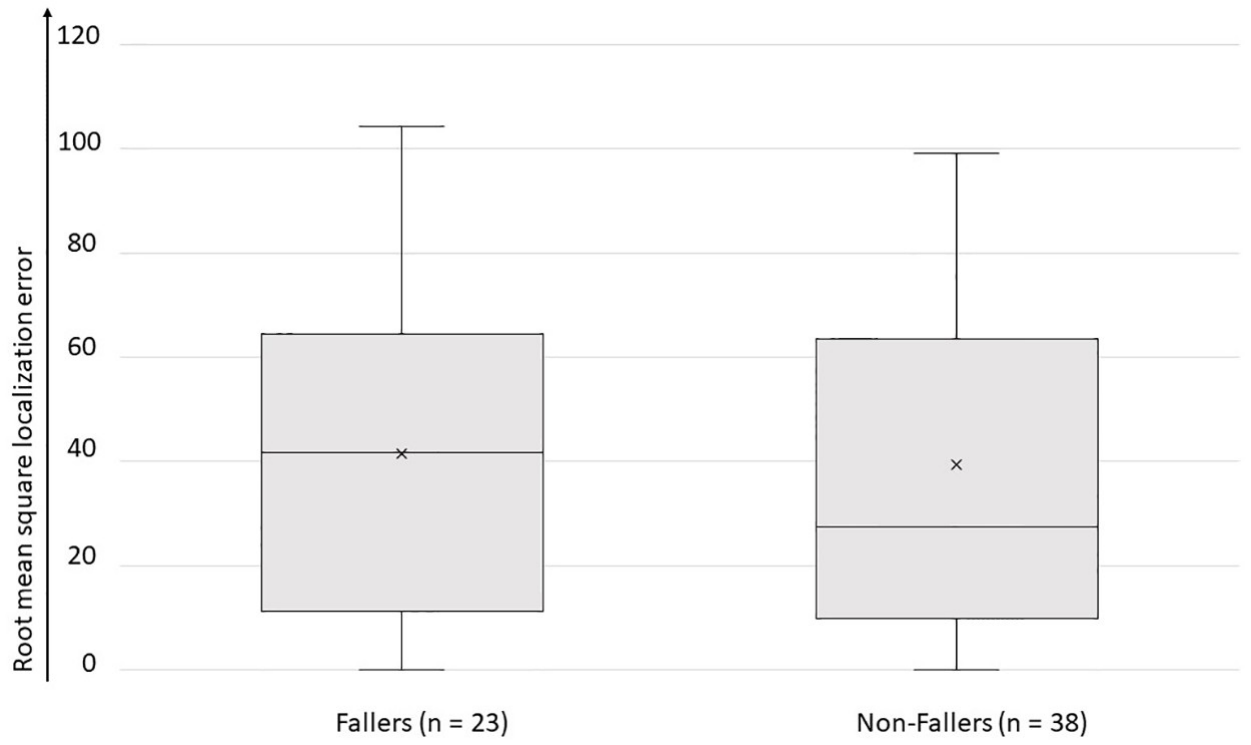
Sound localization test results in best-aided situation. The root mean square localization error is a measurement of patient’s accuracy of sound localization: the higher, the less accurate sound localization. Patients who did not fall in the last year did not have significant better sound localization test results.

Results of the sound localization test are demonstrated in Fig 4. Better sound localization ability was not associated by less falls as there was no significant difference between the RMSE of the fallers (mean RMSE 41.5 ± 29.8) versus the non-fallers (mean RMSE 39.4 ± 33.5).

### Disease/patient’s characteristics and fall risk

**Fig 5:**
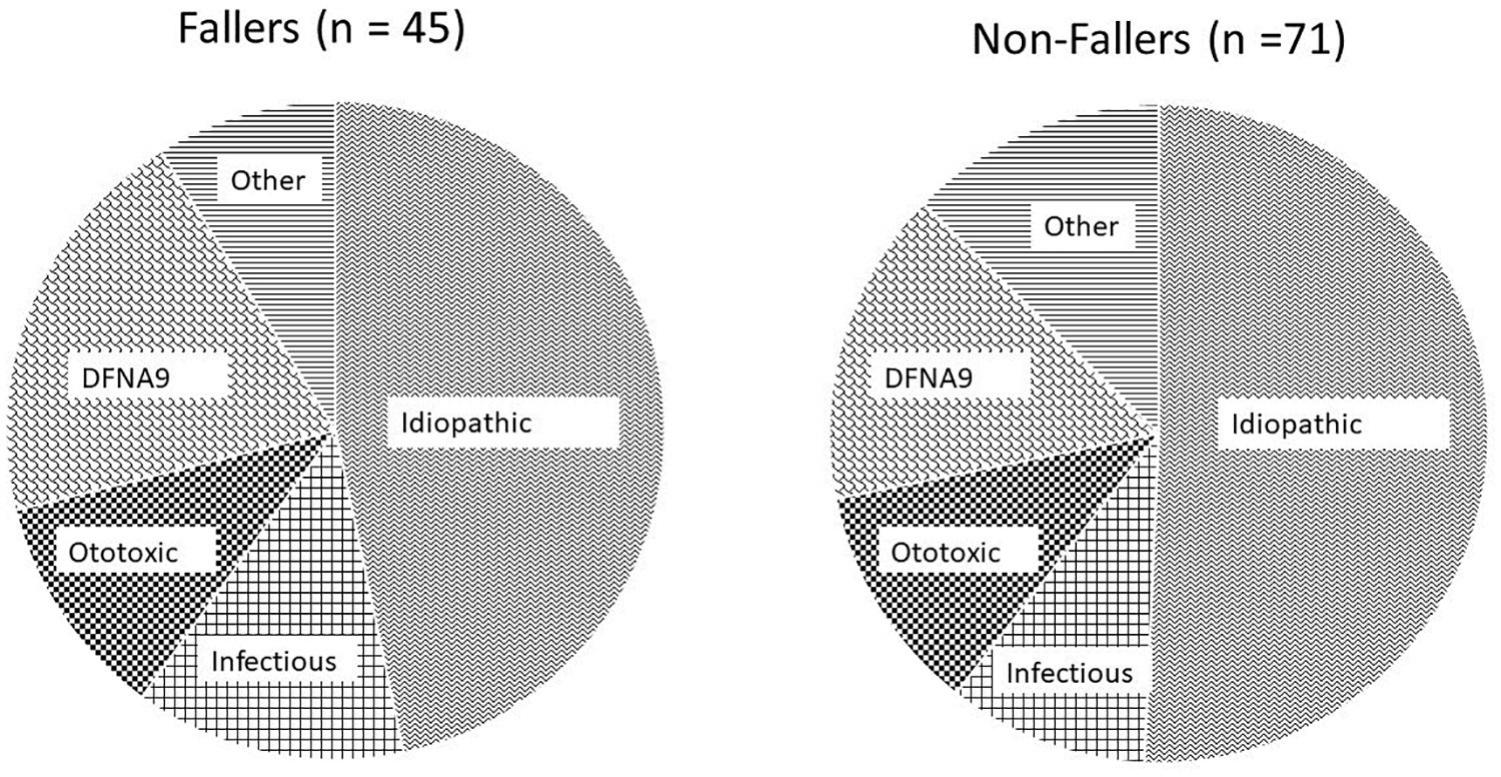
Distribution of etiologies in fallers-group and non-fallers group. ‘Other’ includes Menière’s disease, headtrauma, metabolic, auto-immune, neoplasma and other genetic disorders besides DNFA9 disease.

In Table 3, patient’s and disease characteristics are compared between the faller and non-faller group. Patients who reported falls in the last year tended to be younger and have a shorter disease duration. However, these differences were not significant. Furthermore, no differences in fall incidence was observed between the different etiologies (Fig 5). A clear benefit of sport practice in the prevention of falls could not be identified in the statistical analysis. Likewise, patients with lower body mass indices did not report less falls in the last year.

**Table 3:** Patient and disease characteristics.

	Total patients (n = 119)	Fallers (n = 45)	Non-fallers (n = 71)	Statistics
Sex				
Female	55 (46%)	25 (56%)	29 (41%)	p = 0.1
Male	64 (54%)	20 (44%)	42 (59%)	
Age (mean ± SD, in years)	59.4 ± 12.5	57.8 ± 11.8	62.3 ± 12.2	p = 0.1
Etiology (n, %)				
Idiopathic	59 (50%)	21 (47%)	36 (50%)	p = 0.9
Infectious	13 (11%)	6 (13%)	7 (10%)	
Ototoxic	13 (11%)	5 (11%)	8 (11%)	
DFNA9	20 (17%)	9 (20%)	11 (16%)	
Body-Mass-Index (mean ± SD, in kg/m2)	26.2 ± 4.3	25.8 ± 4.4	26.5 ± 4.3	p = 0.4
Sport practice				
Yes (n, %)	48 (70%)	19 (42%)	28 (40%)	p = 0.8
No (n, %)	21 (30%)	9 (20%)	12 (17%)	
Average hours per week of sport practice (mean ± SD)	2.7 ± 4.2	2.6 ± 5.6	2.8 ± 3.2	p = 0.9
Duration disease (mean ± SD, in years)	12.4 ± 11.3	11.1 ± 11.1	13.6 ± 11.6	p = 0.1

### Symptom questionnaires and fall risk

Forty-two patients (36%) had DHI scores between 30 and 60, indicating a moderate self-perceived handicap. In 40 patients (35%), the self-perceived handicap was severe given the DHI scores greater than 60. BV patients reporting falls in the last year, had significantly higher scores on the DHI and its subscales and the OSQ, see Table 4. After Bonferroni-Holm correction for multiple testing, a significant higher score remained in the faller group on the DHI total scale (p = 0.03), DHI emotional subscale (p = 0.04), DHI physical subscale (p = 0.03) and OSQ (p = 0.01). In contrast, the HADS did not differ significantly between fallers and non-fallers, neither in the Anxiety subscale, nor in the Depression subscale. By means of ROC analyses, a sensitivity of 70% and specificity of 60% was observed to predict falls depending on a DHI > 47, and a OSQ score of > 27.5.

**Table 4:** Comparison of the symptom questionnaires between fallers and non-fallers.

	Fallers (n = 45)	Non-fallers (n = 71)	Statistics	
			Without correction multiple testing	Bonferroni-Holm Correction
DHI total score	54 ± 25	41 ± 23	p = 0.006	p = 0.03
DHI subscore emotional	16 ± 10	11 ± 8	p = 0.009	p = 0.04
DHI subscore physical	17 ± 8	13 ± 8	p = 0.005	p = 0.03
DHI subscore functional	21 ± 10	17 ± 10	p = 0.042	p = 0.1
HADS depression subscale	7.3 ± 5.1	6.2 ± 4.3	p > 0.5	p > 0.5
HADS anxiety subscale	7.1 ± 4.5	6.2 ± 3.7	p > 0.5	p > 0.5
Oscillopsia score	29.8 ± 7.7	24.1 ± 8.2	p = 0.002	p = 0.01

## Discussion

Although BV patients take into account an increased risk of falling and therefore adjust their behavior, this study still identifies a high prevalence of falls: 39% of BV patients reported one or more falls in the preceding year, accompanied by severe injuries (4%). This is consistent with the finding of Sun and colleagues who demonstrated the important impact of BV on quality of life and its economic burden for society [25].

The observed fall prevalence in this study is comparable with previous studies evaluating falls in BV patients. Schniepp and colleagues reported falls in 21 out of 55 (39%) BV patients in the preceding six months [26], Hermann and colleagues reported falls in 11 out of 20 (55%) BV patients since the onset of BV [27]. Results will now be discussed in accordance with the four a priori hypotheses.

### Vestibular tests and fall risk

Residual vestibular function was hypothesized to be higher in the non-fallers compared to the fallers. Contrary to this expectation, none of the vestibular tests (vHIT, calorics, rotatory chair, VEMP) were predictors for fall events. Also, the overall SCC function (captured by all SCC gains during vHIT) was not different between patients experiencing falls and those who did not. Similarly to our findings, Schniepp and colleagues found no correlation between falls and caloric responses in BV patients [26].

In particular, it is somewhat surprising that saccular function did not predict fall risk. Agrawal and colleagues found a greater association between saccular dysfunction (cVEMP amplitudes) and functional impairment (captured by the DHI), compared to canal dysfunction [28]. It should be noted that our entire BV population had bilateral lateral SCC dysfunction, whereas 29% of BV patients had at least a unilateral preserved saccular function. This reflects the fact that the Bárány diagnostic criteria for BV are solely based on the function of the lateral SCC and not on the otolith function. However, previous studies have shown that patients with isolated bilateral otolith dysfunction might exist [29]. It cannot be ruled out that including these patients would alter the results regarding the link between saccular function and fall risk.

Recent studies have shown the wide variety of patterns of vestibular impairment in BV patients [30]. It is conceivable that one single vestibular test evaluating one single vestibular sensor (e.g. vHIT measuring function of each SCC) does not predict fall risk, whereas specific patterns of vestibular impairment could be associated with higher fall risk. For example, the disease specific sparing of the anterior SCC, which is described by previous studies [28, 30] in BV patients with aminoglycosides toxicity, might be associated with a different fall risk depending on otolith function. However, given the wide range of patterns of vestibular impairment, this would lead to too small sample size groups for statistical analyses. Tarnutzer and colleagues demonstrated that hierarchical cluster analysis, which is frequently used in biological sciences, might be suitable to differentiate the diverse pattern of vestibular impairment in BV. Further research should be undertaken to gain more insight in the diverse patterns of vestibular impairment and its link with fall risk.

Nonetheless, we can conclude that single vestibular tests, the overall SCC function (vHIT) and the number of impaired sensors cannot help the clinician identifying BV patients at high risk of falls.

### Hearing performance and fall risk

As the use of external sound sources as spatial landmarks might facilitate gait, we hypothesized that patients with hearing loss are exposed to a higher fall risk. Contrary to expectations, neither the best-aided hearing performance (SPIN), nor the sound localization performance was significantly different between BV patients with and without falls in the preceding year. It is important to note that both test modalities provide complementary information about the hearing status of a patient. While SPIN evaluates binaural daily life hearing performance, the sound localization test enables differentiation of the type of hearing loss (symmetric, asymmetric). Of course, as our study looks at the overall effect of hearing loss on fall risk, it is not excluded that a patient with hearing loss might have a reduced risk of falling when wearing hearing aids. These intra-individual effects of hearing loss should be further studied in prospective cross-sectional studies, as wearing a hearing-aid might be a non-invasive strategy to reduce falls. Interestingly, the study of Hallemans and colleagues observed an improved gait in BV patients when their cochlear implant (CI) was switched on in a room with music. The authors hypothesized that this might be because of the use of external sound sources to maintain balance, or because of direct electrical stimulation of the vestibular system by the CI [12].

### Disease/patient’s characteristics and fall risk

No age difference was observed between fallers and non-fallers. With a mean age of 59 years, this BV population is relatively young. Only few patients were older than 80 years. The rather limited variability of age could be a reason that no significant effect of age is found on the risk of falling. A second potential limitation, because of the relatively young BV population, is that the incidence of severe fall-related injuries, like hip fracture, will be underestimated. Given the central compensation process following the onset of BV, we hypothesized that a shorter disease duration of BV and less sport practice would be risk factors for falling. A tendency towards shorter disease duration in the faller group was observed, however this was not significant. Similarly, one study previously observed no difference in disease duration between BV patients with and without falls in the preceding 6 months [26]. Also sport practice (yes/no and weekly hours of sport practice) did not seem to be different between fallers and non-fallers. Of the patients who practiced sports 48/69 (70%), the majority played sports on a recreational level (e.g. walking, football, …). Surprisingly, even some patients regularly went biking and swimming without problems, reflecting the diversity of experienced symptoms. Only a few patients performed specific vestibular exercises.

Several possible explanations might underly the fact that sport practice did not seem to correlate with fall risk. First, patients able to practice sports are likely those experiencing less subjective symptoms. Therefore, these patients will live a more active life without precautionary measures (walking aid etc.), thereby exposing themselves more frequently to falls compared to those with a sedentary life. Next, it might be possible that specific sport modalities offer a reduction in fall risk, while others don’t (e.g. Tai Chi has been described to be effective in BV patients) [15]. In literature, a wide variety of vestibular rehabilitation exercises and sports have been described. Yet, much uncertainty still exists about the optimal sports and vestibular rehabilitation strategy in BV patients [15]. In other words, the wide variety of practiced sports might be a reason why we did not observe a benefit of sport on fall risk. It is conceivable that some sports, more than other, are preventive for falls.

### Symptom questionnaires and fall risk

Scores on the DHI indicate that the majority of BV patients suffer from a significant decline in quality of life, with 35% of BV patients even perceive their vestibular loss as a severe handicap (DHI scores > 60) [23]. Scores on the DHI and the OSQ both were significantly higher in BV patients experiencing falls in the preceding year. It could be argued that BV patients with higher scores on these questionnaires are more likely to remember the number of experienced falls. While BV patients with lower questionnaire scores, reflecting less subjective symptoms, might be more positively minded and thus underreport experienced falls. However, the HADS did not differ between both groups (fallers versus non-fallers), indicating no difference in anxiety or depression levels. By using ROC analyses, a DHI score > 47 and OSQ score > 27.5 is proposed to predict falls, both having a sensitivity of 70% and specificity of 60% in our population.

Administering the DHI and OSQ before consultation might thus be helpful to recognize BV patients at high risk of falling.

### Risk factors for falls in BV patients according to literature

Schniepp and colleagues investigated predicting factors for falls in 55 BV patients. Similar to our study, age, disease duration, underlying etiology of BV (defined as idiopathic versus secondary BV form) and caloric responses were not found to be different in BV patients reporting falls in the preceding six months versus those that did not fall. Yet, a concomitant peripheral polyneuropathy and increased temporal gait fluctuations during slow walking were risk factors for falls [26]. Furthermore, clinical characteristics and falls were recently evaluated by Hermann et al. in 20 BV patients, of which 55% reported fall since the onset of BV. Significantly higher DHI scores, lower unipedal stance test, higher ataxia numeric scales and greater loss of upwards Dynamic Visual Acuity were observed in the patients with falls. Age was again not different between fallers and non-fallers [27]. On the contrary, Herdmann et al found a younger age to be a risk factor for falling [31]. The authors hypothesized that this might be due to more cautious ambulation in the elderly. Lastly, a study assessing risk factor for falling in a general vestibular population, including 21 BV patients, found an older age to be associated with more falls [9]. Six of the included 21 BV patients experienced recurrent falling in the preceding six months, 2 BV patients reported severe falls with injuries requiring medical attention.

In relation to the results of the present study, it can be concluded that approximately 50% of BV patients reports falls. Although rather rare, BV patients might suffer from severe fall-related injuries like hip and rib fractures. The DHI have repeatedly been demonstrated to be a good predictor for falls, based on our population a cut-off of > 47 would be proposed. Also the Oscillopsia severity questionnaire (OSQ) (score > 27.5) and a unipedal stance test of less than 4 seconds [27] seem indicative for identifying BV patients at risk for falling and can be easily performed at consultation. On the other hand, residual vestibular function, age, hearing, underlying BV etiology and disease duration are not predictive for falls. However, further research into the predictive ability of clinical measures of fall risk or biomechanical stability measures during standing and walking in BV patients could be of vital importance in order to be able to identify those at (high) risk of falling more accurately.

## Conclusion

In this large BV population, the incidence of falls (39%) and severe fall-related injuries (4%) was high. The risk of falling could not be predicted by the hearing status, sound localization performance, duration of disease or sport practice of a BV patient. Moreover, single vestibular tests (vHIT, calorics, rotatory chair and cVEMP) did not seem to correlate with fall risk. The overall residual vestibular function (higher number of impaired vestibular tests) even tended to be lower in patients who did not fall. In contrast, the scores on both DHI and OSQ seemed to be good predictors of the risk of falling.

## Limitations

First, the assessment of the risk of falling was self-reported and retrospective which might result in an underestimation of the true faller rates caused by recall bias. Moreover, the definition of ‘fallers’ was, similar to previous studies in BV patients, chosen to include those BV patients with one or more falls in the last year. Yet, it might be discussed that a single fall can be rather an isolated incident than a true indication of fall risk. Thus, patients with two or more falls in the preceding year might be the patients at a true increased fallrisk instead of those with only one fall event. Therefore, we additionally aimed to identify risk factors for the ‘severe’ fallers experiencing more than one fall, as this might be more sensitive. These statistical analyses, which can be found in detail in the supporting information files (S2), revealed similar results. None of the vestibular tests, or patient’s and disease characteristics were significantly different in the ‘severe fallers’, indicating they were no good predictors of fall risk. Yet, the DHI scores were higher in the severe fallers compared to BV patients with maximum one fall in the past year. The OSQ was not significantly higher in the severe fallers.

Second, the multi-centric nature of the study might impose a disadvantage of center-dependent test modalities. As such, rotatory chair testing was performed in both centers using sinusoidal rotation, yet in UZA at 0.05 Hz while in MUMC at 0.1 Hz. The remaining test protocol was performed in similar conditions. Statistical analyses did not reveal significant differences between the results from both centers. Thirdly, this study focused on the different variables used in the general work-up of a vestibular patient. As a result, no standardized clinical tests determining fall risk (e.g. Timed Up and Go, Tinetti POMA, Functional Gait Assessment, …) or biomechanical gait analysis providing insights into the mechanical control of movement and balance were included in the current study-protocol. These measures could provide additional imperative information in order to predict falls in this patient population by taking the multidimensional character of balance control into account. Lastly, the sample size and characteristics of our patient cohort was determined by the recruitment procedure from the ENT clinic. It is reasonable that recruitment from a neurologic department would result in higher fall percentages as these patients might suffer from more neurological comorbidities (polyneuropathy, CANVAS syndrome, …), while at an ENT clinic the most prominent complaint of BV patients is hearing loss. Nevertheless, to the best of the authors’ knowledge, risk of falling has never been evaluated in BV patients in a study using this large sample size.

## Supporting information

S1 File Patient data information. (SAV)

S2 File Additional statistical analyses with definition of falls as more than one fall in the last year. (DOCX)

S3 File Original questionnaire in Dutch used to assess falls. (DOCX)

S4 File Translated questionnaire (English) used to assess falls. (DOCX)
