# Supplementary material for: Prospective cohort study on the predictors of fall risk in 119 patients with bilateral vestibulopathy
Source: PLoS One. 2020 Mar 9;15(3):e0228768. doi: 10.1371/journal.pone.0228768 (PMC7062241; doi:10.1371/journal.pone.0228768)
Supplement: S2 File — (DOCX) [file pone.0228768.s002.docx]

**Statistical analyses part II**

Based on previous literature of fall risk in BV patients, the definition of ‘fallers’ was chosen to include those BV patients with one or more falls in the last year. However, tt might be discussed that a single fall can be rather an isolated incident than a true indication of fall risk. Thus, patients with two or more falls in the preceding year might be the patients at a true increased fallrisk instead of those with only one fall event. Therefore, we additionally aimed to identify risk factors for the ‘severe’ fallers experiencing more than one fall, as this might be more sensitive. In the subgroup of patients recruited at the UZA, patients were divided in ‘non-fallers’, reporting maximum one fall, and ‘severe fallers’ reporting more than one fall in the preceding year. All statistical analyses were identically performed as described previously with the only difference now being the definition of the ‘non-fallers’ (≤ 1 fall) and ‘severe fallers’ (>1 fall).

As demonstrated below, these statistical analyses revealed similar results.

1. **Vestibular testing**

No significant difference was found between ‘severe fallers’ and ‘non-fallers’ regarding vHIT, caloric and rotatory chair testing. Likewise, cVEMP response did not differ significantly between both groups (chi-squared test, p = 0.5). Also, the total

number of impaired vestibular tests was not significantly different between both groups (Mann-Whitney-U test, p = 0.1)

Table 1A. Results of caloric, rotatory chair and vHIT testing.

|  | Patients with one or no falls (n = 48) | Patients with more than one fall (n = 21) | Statistics | |
| --- | --- | --- | --- | --- |
|  |  |  | Without correction multiple testing | Bonferroni Holm correction |
| **Video HIT** |  |  |  |  |
| gain right lateral | 0.46 ± 0.3 | 0.52 ± 0.3 | p > 0.05 |  |
| gain left lateral | 0.39 ± 0.3 | 0.49 ± 0.2 | p > 0.05 |  |
| gain right posterior | 0.38 ± 0.2 | 0.41 ± 0.2 | p > 0.05 |  |
| gain left posterior | 0.42 ± 0.2 | 0.50 ± 0.2 | p > 0.05 |  |
| gain right anterior | 0.58 ± 0.3 | 0.59 ± 0.2 | p > 0.05 |  |
| gain left anterior | 0.48 ± 0.3 | 0.59 ± 0.3 | p > 0.05 |  |
| sum gain lateral canals | 0.84 ± 0.5 | 1.0 ± 0.4 | p > 0.05 |  |
| sum gain anterior canals | 1.06 ± 0.4 | 1.2 ± 0.4 | p > 0.05 |  |
| sum gain posterior canals | 0.8 ± 0.5 | 0.91 ± 0.4 | p > 0.05 |  |
| sum gain all canals | 2.7 ± 1.3 | 3.1 ± 1.0 | p > 0.05 |  |
| **Calorics** |  |  |  |  |
| sum SPV bilateral bithermal | 3.4 ± 6.0 | 3.7 ± 7.6 | p > 0.05 |  |
| **Rotatory chair test** |  |  |  |  |
| sum SPV bilateral bithermal | 0.08 ± 0.1 | 0.1 ± 0.1 | p > 0.05 |  |

1. **Hearing performance**

Sound localisation performance was not associated with fall risk (mean RMSE in ‘severe fallers’ = 45, in ‘non-fallers’ = 38, Mann-Whitney-U test p = 0.4).

1. **Patient’s characteristics**

As Table 2A demonstrates, no significant differences were shown regarding patient’s and disease’s characteristics between severe fallers and non-fallers.

|  | **Total patients (n = 69)** | **Severe fallers**  **(n = 21)** | **Non-fallers (n = 48)** | **Statistics** |
| --- | --- | --- | --- | --- |
| Sex |  |  |  |  |
| Female | 30 (44 %) | 12 (57 %) | 18 (38 %) | p = 0.1 |
| Male | 39 (56 %) | 9 (43 %) | 30 (63 %) |  |
| Age (mean ± SD, in years) | 59 ± 14 | 56 ± 14 | 61 ± 13 | p = 0.2 |
| Etiology (n, %) |  |  |  |  |
| Idiopathic | 32 | 9 | 23 | p = 0.8 |
| Infectious | 8 | 2 | 6 |  |
| Ototoxic | 3 | 1 | 2 |  |
| DFNA9 | 17 | 7 | 10 |  |
| Body-Mass-Index (mean ± SD, in kg/m2) | 26 ± 5 | 27 ± 5 | 26 ± 4 | p = 0.3 |
| Sport practice |  |  |  |  |
| Yes (n, %) | 48 (70 %) | 14 (67 %) | 34 (71 %) | p = 0.8 |
| No (n, %) | 21 (30 %) | 7 (33 %) | 14 (30 %) |  |
| Average hours per week of sport practice (mean ± SD) | 2.7 ± 4.2 | 1.5 ± 1.9 | 3 ± 4.8 | p = 0.3 |
| Duration disease (mean ± SD, in years) | 13 ± 10 | 11 ± 12 | 14 ± 10 | p = 0.4 |

1. **Symptom questionnaires**

When defining fallers as having more than one fall instead of having at least one fall in the past year, results regarding symptom questionnaires are slightly different. Regarding the DHI questionnaire, both total score and physical subscore remain statistically significantly higher in the ‘severe fallers’ after Bonferroni-Holm correction, while the emotional subscore does not remain significantly higher after correction. Also, the oscillopsia subscore was not significantly different between both groups, whereas this was the case in the first statistical analyses with fallers defined as having at least one fall.

**Table 4**. Comparison of the symptom questionnaires between fallers and non-fallers.

|  | **Severe fallers (n = 21)** | **Non-fallers (n = 48)** | **Statistics** | |
| --- | --- | --- | --- | --- |
|  |  |  | Without correction multiple testing | Bonferroni-Holm Correction |
| DHI total score | 52 ± 29 | 34 ± 24 | p = 0.009 | p = 0.045 |
| DHI subscore emotional | 15 ± 11 | 9 ± 8 | p = 0.03 | p > 0.05 |
| DHI subscore physical | 16 ± 8 | 11 ± 8 | p = 0.01 | p = 0.045 |
| DHI subscore functional | 19 ± 11 | 14 ± 10 | p = 0.04 | p > 0.05 |
| HADS depression subscale | 7.8 ± 4.8 | 6.3 ± 4.6 | p > 0.05 |  |
| HADS anxiety subscale | 7.6 ± 4.3 | 6.0 ± 3.9 | p > 0.05 |  |
| Oscillopsia score | 29.8 ± 7.7 | 24.1 ± 8.2 | p > 0.05 |  |
