# Supplementary material for: Prospective cohort study on the predictors of fall risk in 119 patients with bilateral vestibulopathy
Source: PLoS One. 2020 Mar 9;15(3):e0228768. doi: 10.1371/journal.pone.0228768 (PMC7062241; doi:10.1371/journal.pone.0228768)
Supplement: S3 File — (DOCX) [file pone.0228768.s003.docx]

**Voorbereidende Vragenlijst**

**Naam:**

**Datum:**

**Bent u in het afgelopen jaar gevallen, veroorzaakt door uitglijden of struikelen, waarbij u het evenwicht verloor en terecht kwam op de vloer/grond of een ander lager niveau?**

| Ja [ ] | Nee [ ] |
| --- | --- |

**Indien u wel gevallen bent, hoe vaak is dit gebeurd?**

| Een keer [ ] | Twee keer [ ] | Drie keer of meer [ ] |
| --- | --- | --- |

1. **Waar bent U gevallen?**

| **Binnenshuis:** |  |  |
| --- | --- | --- |
| Op een vlakke ondergrond | Ja [ ] | Nee [ ] |
| Tijdens het opstaan vanuit bed | Ja [ ] | Nee [ ] |
| Tijdens het in of uit een stoel komen | Ja [ ] | Nee [ ] |
| Tijdens het nemen van een bad of douche | Ja [ ] | Nee [ ] |
| Tijdens een bezoek aan het toilet | Ja [ ] | Nee [ ] |
| Tijdens het op- of aflopen van een trap | Ja [ ] | Nee [ ] |
|  |  |  |
| **Ingangen van het huis of in de tuin:** |  |  |
| Tijdens het op- of aflopen van een trap of opstapje | Ja [ ] | Nee [ ] |
| Op een vlakke ondergrond (b.v. het tuinpad) | Ja [ ] | Nee [ ] |
| In de tuin | Ja [ ] | Nee [ ] |
|  |  |  |
| **Buitenshuis:** |  |  |
| Op een voetpad | Ja [ ] | Nee [ ] |
| In de goot | Ja [ ] | Nee [ ] |
| In een openbaar gebouw/ruimte | Ja [ ] | Nee [ ] |
| Bij het uitstappen van een voertuig | Ja [ ] | Nee [ ] |
| Bij iemand anders thuis | Ja [ ] | Nee [ ] |
| Anders, namelijk (voeg uitleg toe): |  |  |
|  |  |  |

1. **Hoe bent u gevallen**

| Ik struikelde | [ ] |
| --- | --- |
| Ik gleed uit | [ ] |
| Ik verloor mijn evenwicht | [ ] |
| Mijn benen konden niet meer | [ ] |
| Ik had het gevoel dat ik flauwviel | [ ] |
| Ik voelde me duizelig | [ ] |
| Ik weet het niet meer | [ ] |

1. **Heeft u verwondingen overgehouden aan deze val of meerdere vallen?**

| Ja [ ] | Nee [ ] |
| --- | --- |

**Indien ‘ja’, wat voor verwondingen waren dit?**

| Blauwe plekken | [ ] |
| --- | --- |
| Schaafwonden | [ ] |
| Gebroken pols | [ ] |
| Gebroken heup | [ ] |
| Gebroken ribben | [ ] |
| Rugpijn | [ ] |
| Anders, namelijk (leg uit): |  |
