# Supplementary material for: Prospective cohort study on the predictors of fall risk in 119 patients with bilateral vestibulopathy
Source: PLoS One. 2020 Mar 9;15(3):e0228768. doi: 10.1371/journal.pone.0228768 (PMC7062241; doi:10.1371/journal.pone.0228768)
Supplement: S4 File — (DOCX) [file pone.0228768.s004.docx]

**Have you fallen in the past year due to slipping or tripping, losing balance and ending up on the floor / floor or another lower level?**

| Yes [ ] | No [ ] |
| --- | --- |
|  |  |

**If you did fall, how often did this happen?**

Once [ ] Two times [ ] Three times or more [ ]

1. **Where did you fall?**

**Indoors:**

On a flat surface Yes [ ] No [ ]

When getting up from bed Yes [ ] No [ ]

When getting in or out of a chair Yes [ ] No [ ]

While taking a bath or shower Yes [ ] No [ ]

During a visit to the toilet Yes [ ] No [ ]

When ascending or descending a staircase Yes [ ] No [ ]

**Entrance to the house or garden:**

When ascending or descending stairs or steps Yes [ ] No [ ]

On a flat surface (e.g. the garden path) Yes [ ] No [ ]

In the garden Yes [ ] No [ ]

**Outdoors:**

On a footpath Yes [ ] No [ ]

In the gutter Yes [ ] No [ ]

In a public building / space Yes [ ] No [ ]

When getting out of a vehicle Yes [ ] No [ ]

At someone else's home Yes [ ] No [ ]

**Other, namely (add explanation):**

1. **How did you fall**

I stumbled [ ]

I slipped [ ]

I lost my balance [ ]

My legs could no longer [ ]

I felt that I was fainting [ ]

I felt dizzy [ ]

I do not know anymore [ ]

1. **Have you sustained injuries from this fall or several falls?**

| Yes [ ] | No [ ] |
| --- | --- |

**If "yes", what kind of injuries were these?**

Bruising [ ]

Scrapes [ ]

Broken wrist [ ]

Broken hip [ ]

Broken ribs [ ]

Backache [ ]

Other, namely
